# Metabolic Syndromes as Important Comorbidities in Patients of Inherited Retinal Degenerations: Experiences from the Nationwide Health Database and a Large Hospital-Based Cohort

**DOI:** 10.3390/ijerph18042065

**Published:** 2021-02-20

**Authors:** Guann-Jye Chiou, Ding-Siang Huang, Fung-Rong Hu, Chung-May Yang, Chang-Hao Yang, Ching-Wen Huang, Jou-Wei Lin, Chao-Wen Lin, Tzyy-Chang Ho, Yi-Ting Hsieh, Tso-Ting Lai, Ho-Min Chen, Pei-Lung Chen, Chuhsing Kate Hsiao, Ta-Ching Chen

**Affiliations:** 1 Department of Medical Education, National Taiwan University Hospital, Taipei 100, Taiwan; jackchiou0920@gmail.com; 2 Department of Ophthalmology, National Taiwan University Hospital, Taipei 100, Taiwan; dshuang@g.ntu.edu.tw (D.-S.H.); fungronghu@ntu.edu.tw (F.-R.H.); chungmay@ntu.edu.tw (C.-M.Y.); chyangoph@ntu.edu.tw (C.-H.Y.); l8513437@gmail.com (C.-W.H.); b91401108@ntu.edu.tw (C.-W.L.); hotchang@ntu.edu.tw (T.-C.H.); ythyth@gmail.com (Y.-T.H.); b91401005@ntu.edu.tw (T.-T.L.); 3 Department of Ophthalmology, College of Medicine, National Taiwan University, Taipei 100, Taiwan; 4 Cardiovascular Center, National Taiwan University Hospital (Yun-Lin Branch), Yunlin 632, Taiwan; jouweilin@gmail.com; 5 Health Data Research Center, National Taiwan University, Taipei 100, Taiwan; homin.chen@gmail.com; 6 Graduate Institute of Clinical Medicine, College of Medicine, National Taiwan University, Taipei 100, Taiwan; paylong@ntu.edu.tw; 7 Graduate Institute of Medical Genomics and Proteomics, College of Medicine, National Taiwan University, Taipei 100, Taiwan; 8 Department of Medical Genetics, National Taiwan University Hospital, Taipei 100, Taiwan; 9 Institute of Epidemiology and Preventive Medicine, National Taiwan University, Taipei 100, Taiwan; ckhsiao@ntu.edu.tw

**Keywords:** inherited retinal degenerations, socioeconomic impacts, Charlson comorbidity index, metabolic syndrome

## Abstract

This study aimed to evaluate the medical and socioeconomic impacts of IRDs using the nationwide health database and a large hospital-based cohort. This retrospective cross-sectional cohort study used data from the nationwide National Health Insurance Research Database (NHIRD). All patients with IRD from January 2012 to December 2016 were selected from the NHIRD and matched with the general population at a ratio of 1:4. All variables, including comorbidities, medications, service utilization, and medical costs, within 1 year from the date of the IRD diagnosis, were analyzed. Disability data were retrieved from the Taiwan Inherited retinal degeneration Project (TIP), a medical center-based database. A total of 4447 and 17,788 subjects from the nationwide database were included in the IRD and control groups, respectively. The Charlson comorbidity index score was higher in the IRD group (0.74:0.52, *p* < 0.001). Yearly visits to the ophthalmology clinic were more frequent in the IRD group (6.80:1.06, *p* < 0.001), particularly to tertiary medical centers (*p* < 0.001). The IRD group showed greater odds ratios (OR) for metabolic syndrome-related comorbidities, including hypertension (OR = 1.18, 95% confidence interval (CI) 1.10 to 1.26) and diabetes (OR = 1.32, 95% CI 1.21 to 1.45), and double the average yearly medical cost (2104.3 vs. 1084.6 USD, *p* < 0.001) and ten times the yearly ophthalmology cost (369.1 vs. 36.1 USD, *p* < 0.001). The average disability level was 54.17% for all subjects. This study revealed the large medical and socioeconomic impacts of IRD on not only patients with IRD, but also their family members and the whole society.

## 1. Introduction

Inherited retinal degenerations (IRDs) are a group of severe retinal degenerative diseases with clinical and genetic heterogeneity. They are estimated to affect approximately 4.5 million people worldwide [1]. Among them, retinitis pigmentosa (RP) is the most common phenotype, with a prevalence of 1 in 3500 live births in Western countries [2] and an even prevalence higher in China [3]. Other common forms of IRDs include cone–rod dystrophy, [4] Leber congenital amaurosis, [5] macular dystrophies (e.g., Stargardt disease, Best vitelliform macular dystrophy), congenital stationary night blindness, and some syndromic diseases with retinopathies, such as Bardet–Biedl syndrome and Usher syndrome. These diseases, caused by degradation of the rod and cone photoreceptor cells, cause progressive, irreversible vision loss. Visual impairment in patients with IRD could start at a young age and thus lead to serious disability for both work and activities of daily living.

To date, studies have rarely focused on the associated comorbidities or socioeconomic impact of IRDs. Frick et al. reported that patients with RP in the USA had a higher prevalence of ocular disorders, including cataract, glaucoma, and other eye disorders [6] and that the annual medical cost of patients with RP was estimated to be 7317 USD higher than that of individuals without RP [6]. A study in Denmark by Bertelson et al. revealed that patients with generalized retinal dystrophy had lower income and civil status compared to healthy controls [7]. However, to the best of our knowledge, no detailed survey has been conducted to explore the causes of increased medical cost for patients with IRDs. Further, no report has detailed the socioeconomic data of Asian patients with IRD.

Taiwan has employed a unique health insurance system called the National Health Insurance (NHI) since 1995. The health insurance premium is calculated according to the monthly income bracket of the insurer, [8] and the insurer can be different from the insured person without income. Owing to the NHI, most enrolled people are protected against unaffordable medical expenses [9]. Every Taiwanese citizen should be enrolled in the NHI by law. According to the annual report of the National Health Insurance Administration, approximately 99.6% of Taiwanese citizens have been enrolled [10]. Therefore, the NHI database can nearly reflect the reality of the medical utilization of the population.

The aims of this study were to explore the medical cost, associated comorbidities, and overall socioeconomic impact of IRDs. The statistical information was retrieved from the databases of the NHI and Taiwan Inherited retinal diseases Project (TIP), a medical-center-based database.

## 2. Materials and Methods

### 2.1. Study Samples

#### 2.1.1. Data Source of the Nationwide Database

This cross-sectional retrospective study was based on the data from Taiwan’s National Health Insurance Research Database (NHIRD). The study protocol was approved by the National Taiwan University Hospital Research Ethics Committee. Due to the anonymity of the data, informed consent was not required.

#### 2.1.2. Study Population, Inclusion Criteria, and Exclusion Criteria

Patients with an IRD-related diagnosis from 1 January 2012 to 31 December 2016 were selected from Taiwan’s NHIRD. Accounting for the shift from the International Classification of Disease, 9th Revision, Clinical Modification (ICD-9-CM) codes to the International Classification of Disease, 10th Revision, Clinical Modification (ICD-10-CM) codes in 2016 in Taiwan, patients documented with ICD-9-CM code 362.7x (hereditary retinal dystrophy) or ICD-10-CM code H35.5x (hereditary retinal dystrophy) were both defined as being diagnosed with IRDs. The documentation of the ICD code was regarded as valid if the code was documented at least twice in the records of outpatient clinics within 1 year or at least once during hospitalization. Study subjects were excluded if the IRD-related diagnosis was documented in 2011 or the years between 2012 and 2015, before the identified year (to prevent duplicate recruitment), if death occurred within 1 year after the first diagnosis, or if the subject had not been enrolled in the NHIRD.

For data analysis, people aged <85 years were divided into 18 groups comprising all 5-year age ranges, and those aged ≥85 years were combined into one group. The age groups were further simplified into three main groups (0–19, 20–64, and 65+) for better demonstration, as shown in Table 1. Patients with IRD were matched to the general population enrolled in the NHI since 2012 in terms of sex, age group, and index year (i.e., control patients were alive at least 1 year after the index date of the matching case).

#### 2.1.3. Study Period

The index date was defined to be the first date of the IRD diagnosis, and index year was defined as the year starting from the index date. The entire study population was observed from the index date to the date of death of the subject or end of the study (31 December 2016). All variables (including comorbidities, medication and medical service utilization, and medical costs) were analyzed within the index year.

### 2.2. Variable Definitions

#### 2.2.1. Urbanization Level of City

The urbanization level of the city in which the population and medical services are located or registered was classified into seven categories in a study by Liu et al. [11]. Level 1 represented the most urbanized city, whereas level 7 was the least urbanized one. Table S1 in the Supplementary shows the details of the criteria of the urbanization level.

#### 2.2.2. Level of Health-Care Utilities

The level of health-care utilities was classified into four levels according to the yearly appraisal by the Ministry of Health and Welfare, Taiwan, which includes medical centers, regional hospitals, local hospitals, and clinics or others. The appraisal level was extracted from the registration data in the NHIRD.

#### 2.2.3. Comorbidities

All 17 Charlson comorbidities were included in the analysis using the enhanced ICD-9-CM and ICD-10 codes [12]. To further understand the conditions of metabolic syndrome and depression, the other four Elixhauser comorbidities, including uncomplicated hypertension, complicated hypertension, obesity, and depression, were added in the analysis [12]. The diagnoses of comorbidities were considered valid if the codes were documented at least twice in the records of outpatient clinics within the index year or at least once during hospitalization.

#### 2.2.4. Medications and Managements of Metabolic Syndrome

The medications commonly used in metabolic syndrome were classified into drugs for diabetes, hypertension, dyslipidemia, and peripheral arterial disease. Each category was subclassified using the Anatomical Therapeutic Chemical Classification. For diabetes, biguanides (A10BA), sulfonylurea (A10BB), α-glucosidase inhibitors (A10BF), thiazolidinediones (A10BG), dipeptidyl peptidase-4 inhibitors (A10BH), glucagon-like peptide-1 receptor agonists (A10BJ), sodium–glucose co-transporter-2 inhibitors (A10BK), glinides (A10BX), and insulins (A10A) were selected. For hypertension, angiotensin-converting enzyme (ACE) inhibitors (C09AA), angiotensin II receptor blockers (C09CA), renin inhibitors (C09XA), α-blockers (C02CA), β-blockers (C07A), calcium-channel blockers (C08CA and C08D), diuretics (C03A, C03B, C03C, C03D, and C03E), other antihypertensive agents (C02A, C02B, C02CC, and C02D), and other anti-hypertensive combinations (C02L, C07BB, C07CB, C09BA, C09BB, C09DA, C09DB, C09DX, and C09XA) were chosen. As for dyslipidemia, we selected only statins (C10AA) and fibrates (C10AB). Cilostazol (B01AC23) was chosen to be analyzed for peripheral arterial disease. Common managements, including examinations (HbA1c, lipid profile, including high-density lipoprotein, low-density lipoprotein, triglyceride, and total cholesterol) and procedures (percutaneous coronary intervention, coronary artery bypass graft surgery, and percutaneous transluminal angiography) were also identified. The records of medications and managements were analyzed if they were prescribed or ordered within the index year.

#### 2.2.5. Medical Cost

The total medical cost was classified into ophthalmology- and other specialty-related costs. In other specialty-related costs, detailed classification was made into outpatient and admission costs. Medications and non-medication costs were also calculated in terms of the ophthalmologic costs and outpatient clinics of other specialties. The currency exchange rate between New Taiwan Dollars and US Dollars (USD, $) was set to be 30:1 in this study.

#### 2.2.6. Specialties of Medical Services

The data of specialties of each medical service or outpatient clinic was extracted from the registration data in the NHIRD. If the medical service was a local hospital or clinic, registration of the main specialty, without that of sub-specialties, was possible.

#### 2.2.7. Disability Level and Percentage Calculations

Because best-corrected visual acuity (BCVA) or visual field (VF) data had not been collected in the NHIRD, to explore the disability level of patients with IRD, we retrieved the information from our clinical database. We, the National Taiwan University Hospital (NTUH), launched the TIP in 2015 for the referral of patients with IRD, to provide clinical service and genetic consultation. We used the disability grading system legislated in our country’s law [13]. Patients were graded by the BCVA or VF data into 15 disability levels, with level 1 indicating the most severe disease and level 15 the least severe one (Table S2 in the Supplementary). Patients with disability levels 1–5, 6–10, and 11–15 or not sufficient for grading were further classified as severely, moderately, and mildly disabled, respectively. To quantify, the average disability percentage was also calculated with the conversion percentage table [14] that is commonly used in our country. Table S3 in the Supplementary shows the conversion percentage of disability.

### 2.3. Statistical Analysis

All data were analyzed with SAS^®^ software version 9.4 (SAS Institute, Cary, NC, USA). In the statistical analysis, the paired *t*-test was used for continuous variables and Mantel–Haenszel test for categorical variables. In ophthalmology-related visits, the chi-square test was used for statistical analysis. All statistical tests were two-tailed, and statistical significance was defined as *p* < 0.05.

## 3. Results

### 3.1. Demographic Data of Study Subjects

**Figure 1:**
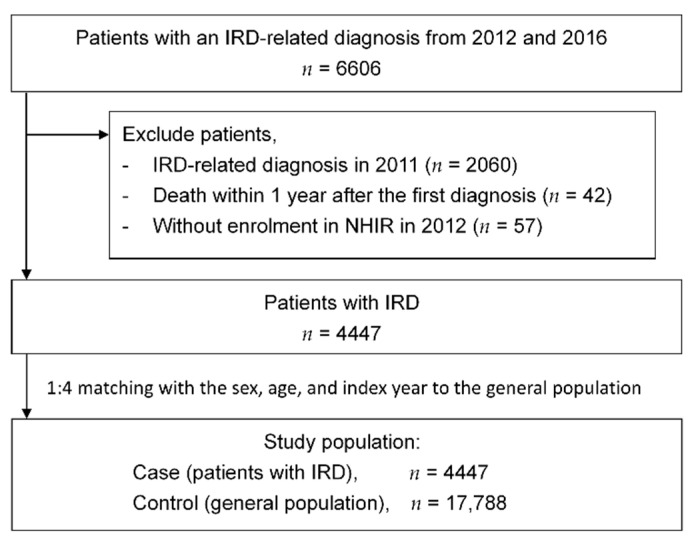
Patient flow diagram. Abbreviations: IRD, inherited retinal degeneration.

According to the NHIRD, the total number of patients with an IRD-related diagnosis from 2012 to 2016 was 6606. We excluded those with an IRD-related diagnosis made in 2011 (*n* = 2060), those who expired within 1 year after the first diagnosis (*n* = 42), and those without enrolment in the NHIRD in 2012 (*n* = 57). For the 4447 included patients with IRD, the general population was matched in terms of the sex, age, and index year at a ratio of 1:4. The study population comprised 4447 patients with IRD and 17,788 people of the general population. Figure 1 shows the patient flow diagram.

Table 1 demonstrates the demographic data. Between a total of 4447 patients with IRD and a control group comprising 17,788 people of the general population, no differences were found in the matched categories, i.e., the age, index date, or sex. No significant difference was found in the geographical distribution between both groups using the urbanization level of cities (*p* = 0.13) and the monthly income bracket according to the NHI (*p* = 0.14). However, the Charlson comorbidity index scores were 0.74 and in the IRD and control groups, showing a significant difference (*p* < 0.001).

### 3.2. Healthcare Utilization and Relationship with the Urbanization Level

**Figure 2:**
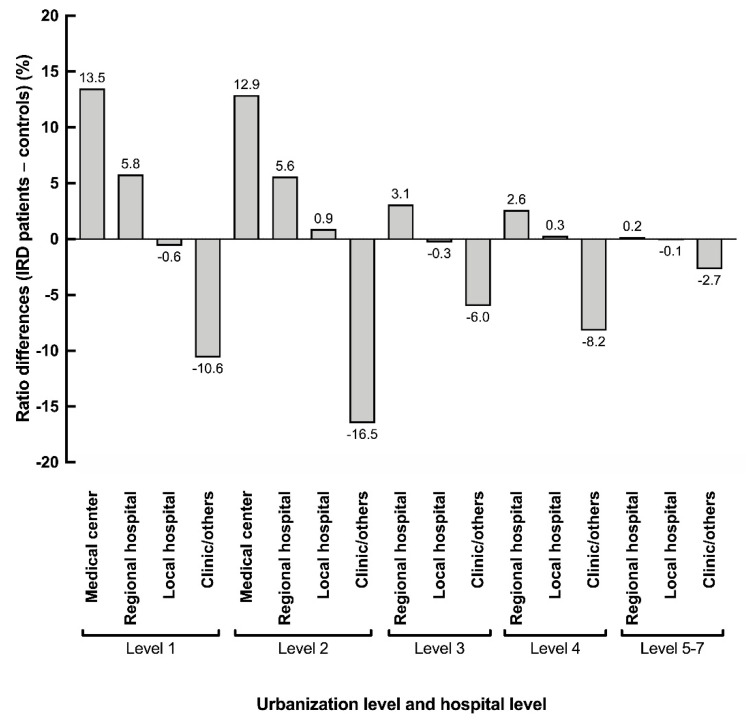
Differences between the patients with inherited retinal degenerations and the control group in the utilization of medical services and the relationship with the city urbanization level.

According to the data retrieved from the NHIRD, the index-year ophthalmology visits from the IRD and control groups were 6.80 (a total of 30,226 times for 4447 patients) and 1.06 (a total of 18,773 times for 17,788 controls) times, respectively, showing a significant difference (*p* < 0.001). Additionally, patients with IRDs utilized significantly more medical resource from the medical centers. Figure 2 illustrated the rate of differences in the hospital level of the visited clinics with the city urbanization level (*p* < 0.001). The increased utilization of medical centers and regional hospitals, rather than private clinics, in the IRD group was consistent at every urbanization level. However, since the medical centers are mostly located in cities, the trend was even more obvious in level−1 and level−2 areas.

### 3.3. Comorbidities Accompanying IRDs

**Figure 3:**
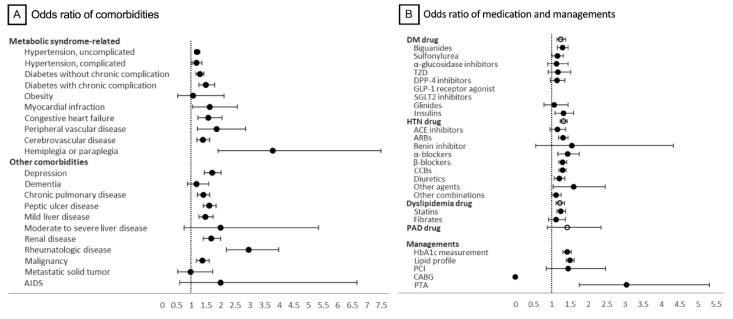
Odds ratio of comorbidities, medications, and managements. The odds ratios were calculated as the odds of IRD patients divided by the odds of control group. The odds were calculated as those with comorbidities in each group divided by those without comorbidities. (**A**) Odds ratios of the metabolic syndrome and other comorbidities were analyzed between the inherited retinal degeneration (IRD) and control groups. (**B**) Odds ratios of the common medications and procedures for metabolic syndrome were analyzed between the IRD and control groups. Abbreviations: AIDS, acquired immune deficiency syndrome; DM, diabetes mellitus; HTN, hypertension; PAD, peripheral artery disease; PCI, percutaneous coronary interventions; CABG, coronary bypass graft surgery; PTA, percutaneous transluminal angiography.

Figure 3A demonstrates the odds ratio (OR) of the comorbidities between the IRD and control groups. We classified these comorbidities into (1) those directly related to metabolic syndromes and (2) others. In the subgroup of metabolic syndrome-related comorbidities, almost every item was more likely to be found in the IRD group than in the control group, including hypertension, combining both uncomplicated and complicated cases (OR = 1.18, 95% confidence interval (CI) 1.10 to 1.26), diabetes with and without chronic complications (OR = 1.32, 95% CI 1.21 to 1.45), myocardial infarction (OR = 1.63, 95% CI 1.03 to 2.57), congestive heart failure (OR = 1.58, 95% CI 1.23 to 2.04), peripheral vascular disease (OR = 1.85, 95% CI 1.20 to 2.86), cerebrovascular disease (OR = 1.38, 95% CI 1.18 to 1.62), and hemiplegia or paraplegia (OR = 3.77, 95% CI 1.91 to 7.48). There was no difference in obesity between the two groups (OR = 1.05, 95% CI 0.52 to 2.11), which was consistent with our clinical impression of few overweight patients with IRD.

In addition to the comorbidities directly related to metabolic syndrome, other diseases warranted our attention. Regarding mental health, there was no difference between the IRD and control groups in dementia (OR = 1.18, 95% CI 0.87 to 1.60), but patients with IRDs were more likely to develop depression (OR = 1.70, 95% CI 1.44 to 2.02). Patients with IRDs were also more likely to have peptic ulcer disease (OR = 1.61, 95% CI 1.41 to 1.84), chronic pulmonary disease (OR = 1.40, 95% CI 1.21 to 1.63), rheumatologic diseases (OR = 2.96, 95% CI 2.20 to 3.98), renal disease (OR = 1.68, 95% CI 1.41 to 2.00), mild liver disease (OR = 1.47, 95% CI 1.25 to 1.74), and malignancy (OR = 1.37, 95% CI 1.16 to 1.61). However, there was no difference between the two groups in moderate to severe liver disease (OR = 2.00, 95% CI 0.75 to 5.34), metastatic tumors (OR = 0.97, 95% CI 0.54 to 1.73), and severe infections, such as acquired immune deficiency syndrome (OR = 2.00, 95% CI 0.60 to 6.65).

### 3.4. Medications for Metabolic Syndromes in Patients with IRD

Since metabolic-syndrome-related diseases developed more frequently in patients with IRD, we further explored the OR of the commonly selected medications and managements for these diseases (Figure 3B). In general, the ORs of medications for diabetes, hypertension, and dyslipidemia were 1.25 (95% CI 1.14 to 1.38), 1.32 (95% CI 1.23 to 1.41), and 1.22 (95% CI 1.12 to 1.34), respectively. All three categories showed significantly increased usage in patients with IRD. However, some subclasses of medications showed no significant difference: ORs of the commonly used biguanides, statins, and ACE inhibitors were 1.29 (95% CI 1.15 to 1.45), 1.24 (95% CI 1.13 to 1.36), and 1.14 (95% CI 0.95 to 1.38), respectively. ACE inhibitors showed no significant difference. Some iconic examinations and managements further proved the increased medical needs in the IRD group, such as the HbA1c measurement (OR = 1.41, 95% CI 1.30 to 1.53) and lipid profile (OR = 1.49, 95% CI 1.39 to 1.60), but the rate of peripheral coronary interventions showed an increased tendency without statistical significance (OR = 1.44, 95% CI 0.84 to 2.47).

### 3.5. Mean Official Medical Cost in Patients with IRD

**Figure 4:**
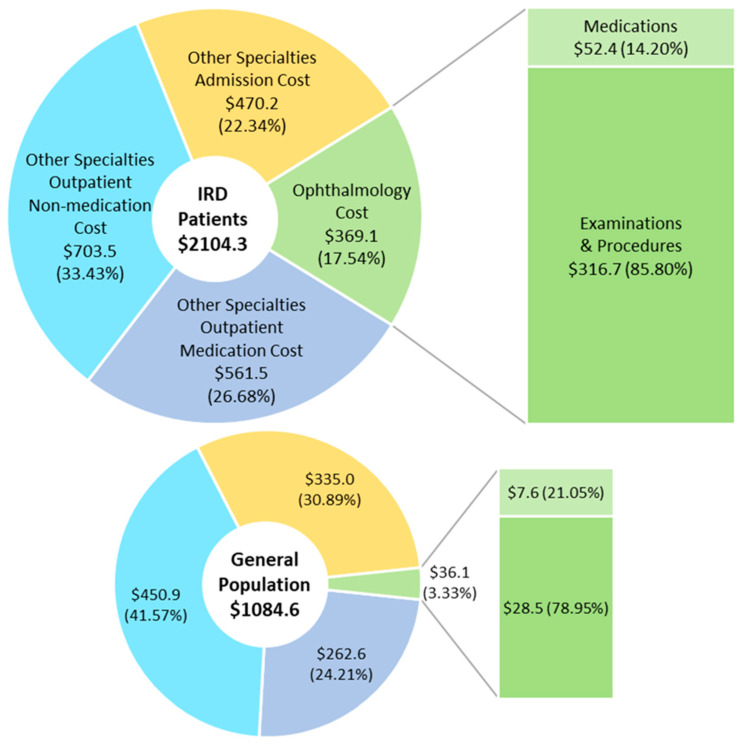
Mean yearly medical costs of patients with inherited retinal degenerations and the general population. Abbreviations: IRD, inherited retinal degeneration.

Yearly medical costs spent within the health insurance system was calculated for both IRD and control groups. Figure 4 illustrates these costs. The average yearly medical costs of the IRD and control groups were 2104.3 and 1084.6 USD, respectively, showing a nearly two-fold (194%) increase for patients with IRD (*p* < 0.001). In the yearly cost for ophthalmological visits, the difference was of nearly 10 times (369.1 USD in the IRD group and 36.1 USD in the control group, *p* < 0.001). Nutrient supplements, such as lutein and vitamins, or genetic testing, are not covered in Taiwan’s official health insurance system. Therefore, the cost listed here mostly covered ophthalmological examinations and consultations rather than medications. In other words, the real cost for patients with IRD in Taiwan could be much higher than the number reported here. In addition to ophthalmology, other specialty-related medical costs were also analyzed. The yearly medical cost was higher in the IRD group than in the control group in both outpatient (*p* = 0.01) and admission (*p* = 0.01) costs.

### 3.6. Increased Medical Needs for Some Specialties in Patients with IRD

**Figure 5:**
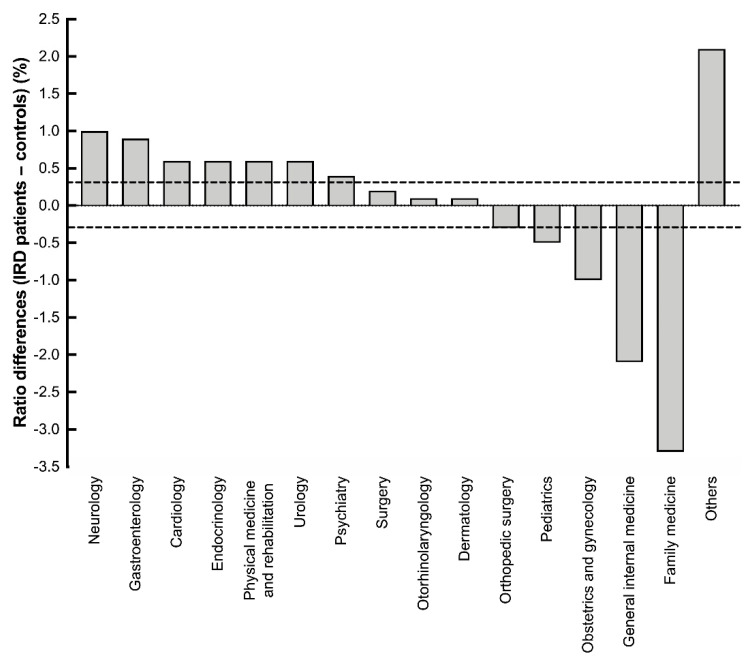
Rate differences in the specialty usage between patients with internal retinal degenerations and the general population. All aforementioned items significantly differed between the groups, with *p* < 0.001. However, we considered the significance to be “clinically meaningful” at a difference of at least 0.3%.

To explore the origins of increased non-ophthalmology medical costs in the IRD group, we further analyzed the usage of different specialties between the IRD and control groups (Figure 5). A difference of 0.3% was set as the clinically meaningful level. As a result, some clinical specialties were more frequently visited by patients with IRD. These specialties were neurology, gastroenterology, cardiology, endocrinology, physical medicine and rehabilitation, urology, and psychiatry, with a difference of 1% (4.3% vs. 3.3%), 0.9% (4.7% vs. 3.8%), 0.6% (6.0% vs. 5.4%), 0.6% (3.9% vs. 3.3%), 0.6% (4.6% vs. 4.0%), 0.6% (2.7% vs. 2.1%), and 0.4% (3.3% vs. 2.9%), respectively. In contrast, visiting rates were lower in family medicine, general internal medicine, obstetrics and gynecology, and pediatrics, with a difference of −3.3% (17.2% vs. 20.5%), −2.1% (11.7% vs. 13.8%), −1% (3.3% vs. 4.3%), and −0.5% (3.6% vs. 4.1%). Nevertheless, all aforementioned items showed statistical significance at *p* < 0.001.

### 3.7. Limitations of Physical Activities and Working Abilities in Patients with IRD

**Figure 6:**
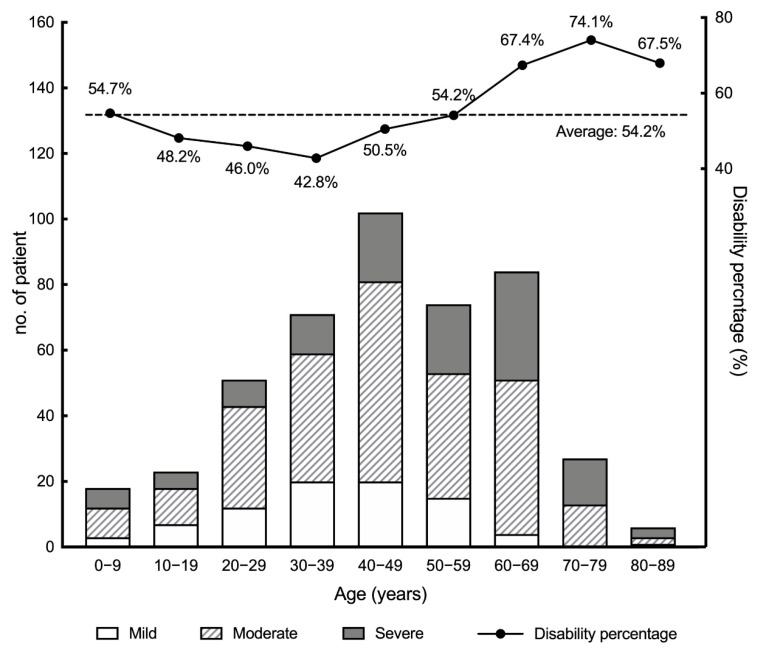
Disability-level distribution and disability percentage of patients with inherited retinal degenerations.

In this present study, we analyzed the first 467 patients recruited in our TIP database who had visited the NTUH in the corresponding time period. Among them, 456 (97.64%) patients had detailed initial BCVA and VF records, available for us to define the official disability level. According to the grading system, 73 (15.63%) patients were not disabled, while the rest of the 383 patients (82.01%) were graded (Figure 6). More patients with IRD who visited our clinic were aged from 20 to 69 years. Among the 456 patients, 251 (55.04%), 123 (26.97%), and 82 (17.98%) patients were moderately, severely, and mildly disabled, respectively. The average disability percentage was calculated to be 54.17% for all subjects, with the least being 42.79% in the age group of 30–39 years and the most being 74.06% in the age group of 70–79 years. From 30s to 80s, the average disability gradually elevated with age. This was reasonable, since the retina degenerates over time in IRDs. However, the average disability was also higher in patients of a very young age: 54.7% and 48.2% in the age groups of 0–9 and 10–19 years, respectively). It might originate from some early-onset phenotypes, such as Leber congenital amaurosis and early-onset cone–rod dystrophy. The findings could support the possible linkage, where metabolic syndrome-related comorbidities may be related to the limitations of physical activities and working abilities in patients with IRD.

## 4. Discussion

According to Bertelsen et al.’s study in Denmark, [7] the socioeconomic status of patients with IRD was significantly lower compared to the general population. However, our results (Table 1) showed no significant difference in the monthly income between the IRD and control groups. In Taiwan’s NHI, a person’s insurance could be paid under another insurance applicant when the person has no or a low income. Since the employment rate was significantly lower among patients with an IRD than among controls, as reported by Bertelsen et al., they were probably enrolled in the NHI under another insurance applicant’s name, who could pay the premium for the insured patient. Therefore, the monthly income bracket according to the NHI may not reflect the true income of patients with IRD themselves but of their next of kin.

Significantly higher Charlson Comorbidity Index scores were found in our study in patients with IRD. To the best of our knowledge, this is the first study on all kinds of comorbidities accompanying IRDs. Previously, Frick et al. compared the Charlson Comorbidity Index score between the RP and non-RP groups but found no difference [6]. A study by Crewe et al. showed the comorbidity rate of hospital admission, which would possibly be biased, because most comorbidities are managed at outpatient clinics [15]. The strength of our study was that we included all data in inpatient and outpatient records from the nationwide health database NHIRD.

Among all kinds of comorbidities, chronic diseases, such as metabolic syndrome-related diseases, mainly differed between the IRD and control groups rather than in terms of trauma or accidents, consistent with our clinical observations. Most Taiwanese live in urban areas with family members, and the family style is usually core or extended family, for example, a family with three generations. Therefore, patients with IRD are often well-protected and accompanied. This may prevent them from accidents and trauma but may also further limit their physical activities.

Metabolic-syndrome-related diseases seemed to be prevalent as comorbidities accompanying IRDs. Higher ORs were found for nearly all kinds of diseases, including hypertension, diabetes, and other possibly related diseases or syndromes, except for obesity. Previous studies by St-Onge, [16] Hadaegh, [17] and Suliga et al. [18] showed that metabolic syndrome could also develop in people with normal weight or slightly overweight body mass index. Nevertheless, the possible cause of these diseases should be explored. It is interesting that not only did the analysis of main categories of medications for treating metabolic syndrome show increased odds, most of the subclasses showed a similar tendency, with no statistical significance. This phenomenon may reflect that the selection principle for the medication was not significantly affected by the underlying disease of IRDs.

The higher rate of metabolic syndrome in the IRD group may be attributed to the high disability rate and activity limitations. Whitson et al.’s study suggested that people with vision impairment had greater odds of mobility disability [19]. Patel et al.’s study also suggested that those with visual impairment would have lower mobility activities compared to those without [20]. However, both authors acknowledged that self-reported data were the limitation of their studies [19,20], particularly in the dementia group [20]. In contrast, we chose to gain disability data on an objective basis, and 84% of our patients with IRD (383 of 456) were officially in the decreased “disability status”, with an average of 54.17% loss of working disability being disclosed. We assumed that working disability was directly proportional to mobility limitations and inferred that there may be mobility limitations in patients with IRD. Moon et al.’s study suggested that the risk of metabolic syndrome was increased in patients with low muscle mass despite normal body mass index, and this could be the reason why the odds of metabolic syndrome were greater among these patients [21].

Our results also indicated greater odds of depression in patients with IRD. Similarly, a study by Kim et al. revealed a higher depressive status rate in patients with RP [22] and another by Chaumet-Riffaud et al. showed similar results [23]. A systemic review and meta-analysis also revealed that the prevalence of depression was higher among patients with eye disease than among controls [24]. Kim et al. also showed that higher rates of patients with RP reported moderate to severe stress compared to controls [22]. Although the *Helicobacter pylori* infection rate and use of non-steroidal anti-inflammatory drugs were not collected in our study, a higher stress level probably contributed to the higher odds ratio of peptic ulcer disease [25].

Our results revealed no difference in the distribution of the city urbanization level between the two groups. However, patients with IRD needed more medical care in medical centers compared to the general population, particularly for ophthalmology. The trend was even more obvious in cities, possibly because medical centers are usually located in cities. The significantly higher mean times of visiting ophthalmology clinics in 1 year in the IRD group reflected the culture and national conditions of our country, implying that medical accessibility and density are high in Taiwan. These patients could easily access medical services when necessary.

Among all specialties, the higher visiting rates for neurology, cardiology, endocrinology, physical medicine and rehabilitation, psychiatry, and gastroenterology were consistent with greater odds of metabolic syndrome-related diseases and depression or stress. The lower visiting rates for family medicine and general medicine were correlated with the lower visiting rates of local clinics and local hospitals. Although the age and sex were matched, the visiting rates for pediatrics and obstetrics and gynecology were significantly lower in the IRD group than in the control group. A previous study showed that the marital rate among patients with IRD was lower [7]. The low marital rate and inheritable nature of IRD were thought to be the causes of the lower visiting rates for obstetrics and gynecology, while parents focusing on ophthalmologic disorders probably caused the lower visiting rate for pediatrics. Some specialties had a similar usage between the IRD group and normal population, such as surgery and orthopedic surgery, which may reflect the good family support system in Taiwan, and no obvious increase of trauma or accident in patients of IRD. Regarding the age on the index year, about 25% of IRD patients were aged more than 65 years. This may also reflect that IRD patients tended to seek medical help when they had severely visual impairment and other systemic comorbidities.

Our results from the nationwide health database, NHIRD, demonstrated that the yearly medical cost of a single patient with IRD was nearly twice (194%) that of a person of the general population and that the ophthalmology-related cost was approximately 10 times higher among patients with IRD. Many studies also proved that conditions of visual impairment, blindness, or some kinds of IRDs, such as RP and Stargardt disease, were correlated with higher medical service utilization or medical costs [6,15,26,27]. In this official database of the NHIRD, most ophthalmology-related costs comprised examinations and procedures rather than medications. Nutrient supplements, such as lutein and vitamins, and some complementary and alternative medications, such as Chinese herbs, are not covered in our health insurance and were therefore not officially recorded. Furthermore, genetic testing is not covered in Taiwan’s official health insurance system. Therefore, the “true” total ophthalmology-related cost in patients with IRD could be far more than 10 times that of the general population.

To evaluate the socioeconomic impact of a disease, both the disease-related cost and total medical needs should be calculated. Our data revealed that both IRD and control groups spent over 80% of their medical cost in specialties other than ophthalmology (and even over 95% in the control group), and over 60% in outpatient clinics. A concomitant finding was that nearly 45% of the outpatient cost of other specialties was incurred by patients with IRD for medications, which is greater than the 37% spent in the control group. Our data of the higher ORs of comorbidities could support this finding. More comorbidities to be treated raises the medical costs in the outpatient clinics.

Our study suggested that the mean annual medical cost of each patient with IRD was nearly twice than that of the control, but the difference was merely 1019.7 USD. Frick et al. reported that the annual medical costs of patients with RP were estimated to be 7317 USD higher than those without RP. [6] The difference may because of the relative affordability of medical services in Taiwan under the support of the NHI [9], meaning the medical cost would be much lower compared to the US or European countries. Taiwan is famous for the extremely high cost-to-performance ratio of its medical service, and the nationwide health insurance system. In addition, many other costs were not covered by the NHI, such as self-paid medications or medical equipment, or costs of alternative medicine. The medical costs of these patients were probably underestimated.

Another uncountable medical cost was generated implicitly from the support of the patient’s family. Based on our own experience, most patients with IRD had to rely on a family member’s support to reach the clinic. Influenced by the Eastern culture, the family’s support and connection between family members in Taiwan are usually strong, which could lower the chances of accidents and trauma for patients with IRD but lead to additional costs from the loss of a partner’s productive forces, possibly further limiting the physical activities of these patients.

**Figure 7:**
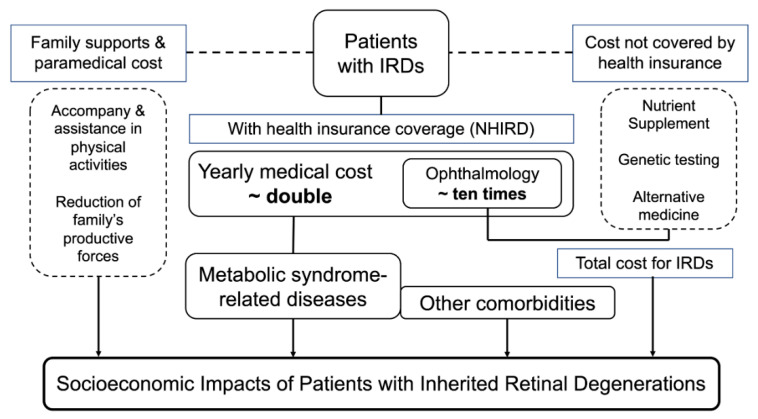
A brief summary of medical and socioeconomic impacts of inherited retinal degenerations. Abbreviations: IRD, inherited retinal degeneration.

After summarizing all the explicit and implicit costs and considering the less expensive medical services in Taiwan, the total medical cost should be far higher than it seemed to be. Figure 7 summarizes the socioeconomic impacts of IRD that we found in the present study.

There were limitations to this study. First, although we retrieved the data of a total of 22,235 people from the nationwide database, this was a retrospective cross-sectional cohort study. A future, prospective study may further strengthen the findings. Second, the disability grading level and potential reduction in physical activities were inferred from our hospital-based database rather than the nationwide NHIRD, because no detailed BCVA or VF data could be extracted from the NHIRD. However, since our TIP indiscriminately recruited patients with IRD across Taiwan, encompassing approximately one-fourth of the patients with IRD in Taiwan, the disability trend of patients with IRD should be the same as, or at least similar to, the nationwide NHIRD results. Third, the quantitative relationship between the working disability and the limitation of physical mobility is unclear and warrants further study in the near future. Fourth, as the clinical manifestation of different IRDs varies greatly, the accuracy of the diagnosis and coding is critical for the accuracy of the study results. We could not validate the genetic diagnosis through NHIRD, but we could be confident of the clinical and genetic diagnosis in patients recruited in our TIP cohort, which encompassed approximately one-fourth of the patients with IRD in Taiwan. Finally, there are still some other important disorders that may cause blindness, such as cataract, glaucoma and diabetic retinopathy, but we could not include all the yearly medical costs related to these disorders into our present study. One previous report also revealed increased healthcare costs associated with progressive diabetic retinopathy [28]. Further studies may focus on the comparison of relative socioeconomic burden among different kinds of ocular disorders. 

In summary, this was a nationwide unbiased database study to disclose the impact of IRD on the patient’s socioeconomic status from the basis of comorbidities. To the best of our knowledge, this is the first study using the nationwide database to investigate the relationship between IRD and the greater odds of metabolic syndrome-related diseases and quantified disability level. The results also reflected the family model in Eastern societies and Western cultures. Our results demonstrated that large medical and socioeconomic burdens existed for not only patients with IRD, but also their family members and the whole society. Novel therapeutic methods, including gene therapies, will be valuable to treat these patients or at least maintain their life quality with few adverse effects [29,30,31,32]. With the ongoing studies on gene therapies and the other novel therapeutic methods, these patients could be treated in the near future, and the socioeconomic burden could be relieved.

## 5. Conclusions

To the best of our knowledge, this is the first study using the nationwide database to investigate the relationship between IRD, greater odds of metabolic-syndrome-related diseases and quantified disability level. The results also reflected the family model in Eastern societies and Western cultures. Our results demonstrated that large medical and socioeconomic burdens existed, not only for patients with IRD but also their family members and the whole society. Novel therapeutic methods, including gene therapies, will be valuable to treat these patients, or at least maintain their life quality with few adverse effects.

## Figures and Tables

**Table 1:** Demographic Data of the Study Cohort.

	IRD (*n* = 4447)	Control (*n* = 17,788)	*p* Value
Age on the index date			
Mean (SD)	51.8 (18.1)	51.7 (18.2)	-
Median (Q1, Q3)	54 (40, 64)	54 (40, 64)	
Age			
0–19, *n* (%)	241 (5.42)	968 (5.44)	-
20–64, *n* (%)	3100 (69.71)	12454 (70.01)	
65+, *n* (%)	1106 (24.87)	4366 (24.54)	
Sex			
Male, *n* (%)	2079 (46.75)	8316 (46.75)	-
Female, *n* (%)	2368 (53.25)	9472 (53.25)	
Urbanization level of the registered city			
Level 1, *n* (%)	1432 (32.20)	5388 (30.29)	0.1323
Level 2, *n* (%)	1363 (30.65)	5637 (31.69)	
Level 3, *n* (%)	727 (16.35)	2986 (16.79)	
Level 4, *n* (%)	588 (13.22)	2281 (12.82)	
Level 5, *n* (%)	69 (1.55)	314 (1.77)	
Level 6, *n* (%)	130 (2.92)	601 (3.38)	
Level 7, *n* (%)	138 (3.10)	581 (3.27)	
NHI monthly income bracket			
<666.7 USD, *n* (%)	1244 (27.97)	4780 (26.87)	0.1426
666.7–1000 USD, *n* (%)	1619 (36.41)	6809 (38.28)	
1000–1333.3 USD, *n* (%)	525 (11.81)	2147 (12.07)	
1333.3–1666.7 USD, *n* (%)	479 (10.77)	1821 (10.24)	
1666.7+ USD, *n* (%)	580 (13.04)	2231 (12.54)	
Charlson comorbidity index			
Mean (SD)	0.74 (1.41)	0.52 (1.21)	<0.001
Median (Q1, Q3)	0 (0, 1)	0 (0, 1)	
Ophthalmology visit times from the index date to 1 year later			
Mean (SD)	6.80 (5.40)	1.06 (2.69)	<0.001
Median (Q1, Q3)	5 (3, 9)	0 (0, 1)	

IRD, inherited retinal degeneration; SD, standard deviation; NHI, national health insurance.

## Data Availability

Restrictions apply to the availability of these data. Data was obtained from Taiwan’s National Health Insurance Research Database (NHIRD) and are available with the permission of NHIRD.

## Supplementary Materials

The following are available online at https://www.mdpi.com/1660-4601/18/4/2065/s1, Table S1: Criteria of the city urbanization level; Table S2: Criteria of disability level; Table S3: Disability percentage conversion table.
