# Supplementary material for: Metabolic Syndromes as Important Comorbidities in Patients of Inherited Retinal Degenerations: Experiences from the Nationwide Health Database and a Large Hospital-Based Cohort
Source: Int J Environ Res Public Health. 2021 Feb 20;18(4):2065. doi: 10.3390/ijerph18042065 (PMC7923804; doi:10.3390/ijerph18042065)
Supplement: Supplementary file 1 [file ijerph-18-02065-s001.pdf]

Table S1. Criteria of the city urbanization level.

Urban Level	Description	Population density	College or above educational levels	People over 65 years old	Agriculture workers	Physicians density
Level 1	Highly urbanized city	1	1	5	7	1
Level 2	Moderately urbanized city	2	2	6	6	2
Level 3	Boomtown	3	3	7	5	4
Level 4	Normal town	4	4	3	4	3
Level 5	Aging city	6	5	1	2	7
Level 6	Agricultural city	7	7	2	1	5
Level 7	Remote town	5	6	4	3	6

Reference: Liu, C.-Y.; Hung, Y.-T.; Chuang, Y.-L.; Chen, Y.J.; Weng, W.S.; Liu, J.S. Incorporating development stratification of Taiwan townships into sampling design of large scale health interview survey. *J. Health Manag.* **2006**, *4*, 1–22.

Table S2. Criteria of disability level

<table>
<tr><th rowspan="2"></th><th colspan="3">Description</th><th rowspan="2">Level</th></tr>
<tr><th>Bilaterally……</th><th>Unilaterally blind plus another eye……</th><th>Unilaterally……</th></tr>
<tr><td rowspan="10">Visual acuity disability</td><td>Totally blind</td><td></td><td></td><td>2</td></tr>
<tr><td>Weakened to less than 0.02 but have not reached totally blind</td><td>Weakened to less than 0.02 but has not reached totally blind</td><td></td><td>3</td></tr>
<tr><td></td><td>Weakened less than 0 .06</td><td></td><td>4</td></tr>
<tr><td>Weakened to less than 0.06</td><td></td><td></td><td>5</td></tr>
<tr><td></td><td>Weakened to less than 0.1</td><td></td><td>6</td></tr>
<tr><td>Weakened to less than 0.1</td><td>Weakened to less than 0.4</td><td></td><td>7</td></tr>
<tr><td></td><td></td><td>Those whose one eye is blind</td><td>8</td></tr>
<tr><td></td><td></td><td>Weakened to less than 0.02 but have not reached totally blind</td><td>9</td></tr>
<tr><td>Weakened to less than 0.4</td><td></td><td>Weakened to less than 0.06</td><td>10</td></tr>
<tr><td></td><td></td><td>Weakened to less than 0.1</td><td>11</td></tr>
<tr><td rowspan="2">Visual field disability</td><td colspan="3">Those whose both eyes are involved in hemiscotosis, narrow or deformation vision</td><td>10</td></tr>
<tr><td colspan="3">Those whose one eye is involved in hemiscotosis, narrow or deformation vision.</td><td>14</td></tr>
</table>

Note:

Disability examination of visual acuity:

1. The measurement of "visual acuity":
   A. Based on corrected visual acuity with the application of C shape eye chart, but those who cannot be corrected or are involved in aniseikonia should be measured by one the naked eyesight.
   B. The measurement of visual acuity disability shall pass Malingering examination.
2. "Blindness" includes eyeball losing or extraction, or visual acuity including no light perception, hand motion within 1 m, and counting fingers within 5 cm.
3. If one has two of the "Visual acuity disability", "Visual field disability", and "Regulating or moving disability" (not listed here), the grading of disability could be increased according to regulation, but the highest grading is that both eyes could not exceed the second level, and one eye could not exceed the eighth level with the exception of eyelid defect disability.

Disability examination of visual field:

1. On the judgment of visual field, those whose visual field is decreased to less than 60% of normal visual field measured at the optotype diameter 1 cm under daylight and eight-point vision angle are called vision deformation. Scotoma bases on absolutely scotoma, and the comparative scotoma is not available on this list
2. The determination of "Visual field disability" should be based on the "Fundus or optic disc image centered on the optic nerve and the macula lutea" and the "Visual field test" within the last three months. Confirmed diagnosis in Malingering examination is also required.

Reference: Ministry of Labor, Executive Yuan, Taiwan. *Labor Insurance Disability Benefit Payment Standards*; Council of Labor Affairs of Executive Yuan: Taipei, Taiwan, 2015.

Table S3. Disability percentage conversion table

Level	Disability percentage	Level	Disability percentage
N/A	0%	8	65.52%
15	7.69%	7	69.21%
14	15.38%	6	76.9%
13	23.07%	5	84.59%
12	30.76%	4	92.28%
11	38.45%	3	100%
10	46.14%	2	100%
9	53.83%	1	100%

Abbreviation: N/A, not applicable

Reference: Tseng, L.H. *詳解損害賠償法 [Explaining the Law of Damages in Detail]*; San Min Book Co., Ltd.: Taipei, Taiwan, 2008; p. 346.
